# Anticipating implementation: A mixed methods study of future prescribing determinants within an ongoing trial of clinical effectiveness

**DOI:** 10.1186/s43058-026-00969-0

**Published:** 2026-05-29

**Authors:** Jennifer Kononowech, Allison Ranusch, Anne E. Sales

**Affiliations:** 1 Center for Clinical Management Research, VA Ann Arbor Healthcare System, 2800 Plymouth Rd, Ann Arbor, MI 48109 United States; 2 Sinclair School of Nursing and Department of Family and Community Medicine, University of Missouri, Columbia, MO 65211 United States

**Keywords:** Implementation science, Clinical trial, Diabetic kidney disease, Prescribing, Department of veterans affairs, Cooperative studies program

## Abstract

**Background:**

The Department of Veterans Affairs Cooperative Studies Program (CSP) added implementation science teams to select trials to accelerate the uptake of research evidence into practice. CSP study #2008, “Pentoxifylline in Diabetic Kidney Disease”, evaluates whether adding pentoxifylline to usual care reduces the incidence of end-stage renal disease and mortality in patients with diabetic kidney disease. This implementation science sub-study aims to develop a VA-wide implementation plan by identifying key implementation outcomes, identifying and interviewing key stakeholders, and identifying critical barriers and facilitators to implementing findings.

**Methods:**

Using an exploratory sequential mixed methods approach in this hybrid type 1 design, we conducted semi-structured interviews with nephrologists, primary care physicians, and pharmacists from ten VA facilities that were enrolling Veterans in the CSP 2008 clinical trial. The interviews were analyzed using the Tailored Implementation in Chronic Disease (TICD) framework. Findings informed a web-based survey distributed to nephrologists, primary care physicians, and pharmacists from 15 additional participating facilities.

**Results:**

Interviews identified five facilitators: 1) providers are willing to prescribe an older medication; 2) providers are not concerned about the side effects of pentoxifylline; 3) providers believe that pentoxifylline is inexpensive; 4) providers report widespread comfort with off-label prescribing; and 5) providers state they would be comfortable prescribing pentoxifylline if colleagues they trust recommend it. Survey data confirmed that providers are not concerned with prescribing an older medication, but there were mixed responses about off-label prescribing.

Barriers included: 1) concerns about possible drug interactions; 2) providers self-identified as late adopters; 3) concerns about polypharmacy; 4) prescribers prefer newer drugs; and 5) providers want ample evidence that pentoxifylline works for this indication. Survey data confirmed all the identified barriers except for providers identifying as late adopters.

**Conclusions:**

Interview data provided early evidence of facilitators that may support pentoxifylline adoption if the trial shows positive results. Survey data reinforced and broadened these insights across a larger clinician sample. Together, these findings will guide development of targeted implementation strategies to support effective and timely dissemination of trial findings across the VA health system.

**Trial registration:**

The CSP 2008 study ClinicalTrials.gov ID is NCT03625648.

**Supplementary Information:**

The online version contains supplementary material available at 10.1186/s43058-026-00969-0.

**Table:** 

Contributions to the literature
• Our findings highlight the importance of identifying facilitators and barriers to implementation as clinical trials are underway to enable more rapid implementation of research findings.
• Findings from this study support prior work showing that there are several barriers to changing provider prescribing practice and suggest that multiple implementation strategies will need to be designed to support change.
• Our work provides an example of using an exploratory sequential mixed methods approach to encompass a broader sample to confirm qualitative findings and support development of implementation strategies, in the context of highly prospective implementation planning.

## Background

Ensuring that research findings are disseminated and implemented in a timely manner is a priority for implementation scientists [1, 2]. However, studies show that it can take considerable time to get research evidence into practice [3].

The Department of Veterans Affairs (VA) Cooperative Studies Program (CSP) is a research enterprise under the Office of Research and Development (ORD) that plans and conducts large multicenter clinical trials and epidemiological studies in the VA [4]. Recognizing the need to accelerate the uptake of research findings, CSP incorporated implementation science teams into a small number of trials to plan for dissemination and implementation should the trial hypothesis be supported [5, 6]. This approach aligns with principles of a hybrid type 1 design, focusing primarily on testing the clinical intervention while prospectively assessing implementation determinants to accelerate uptake if the intervention proves beneficial [7, 8]. Although planning for implementation prior to determining effectiveness may appear premature, this approach requires relatively modest additional resources and will enable rapid translation of findings into practice, which addresses the well-documented delays in evidence uptake.

Cooperative Studies Program #2008 (CSP 2008), “Pentoxifylline in Diabetic Kidney Disease” is an ongoing randomized controlled trial that is testing the hypothesis that pentoxifylline added to usual care leads to a reduction in the incidence of end-stage renal disease and mortality in type 2 diabetic patients with diabetic kidney disease [9]. Pentoxifylline has been used for decades to treat peripheral vascular disease [10]. There are several examples of medications developed for different indications that were later repurposed for new uses and tested through clinical trials [11, 12].

**Fig. 1:**
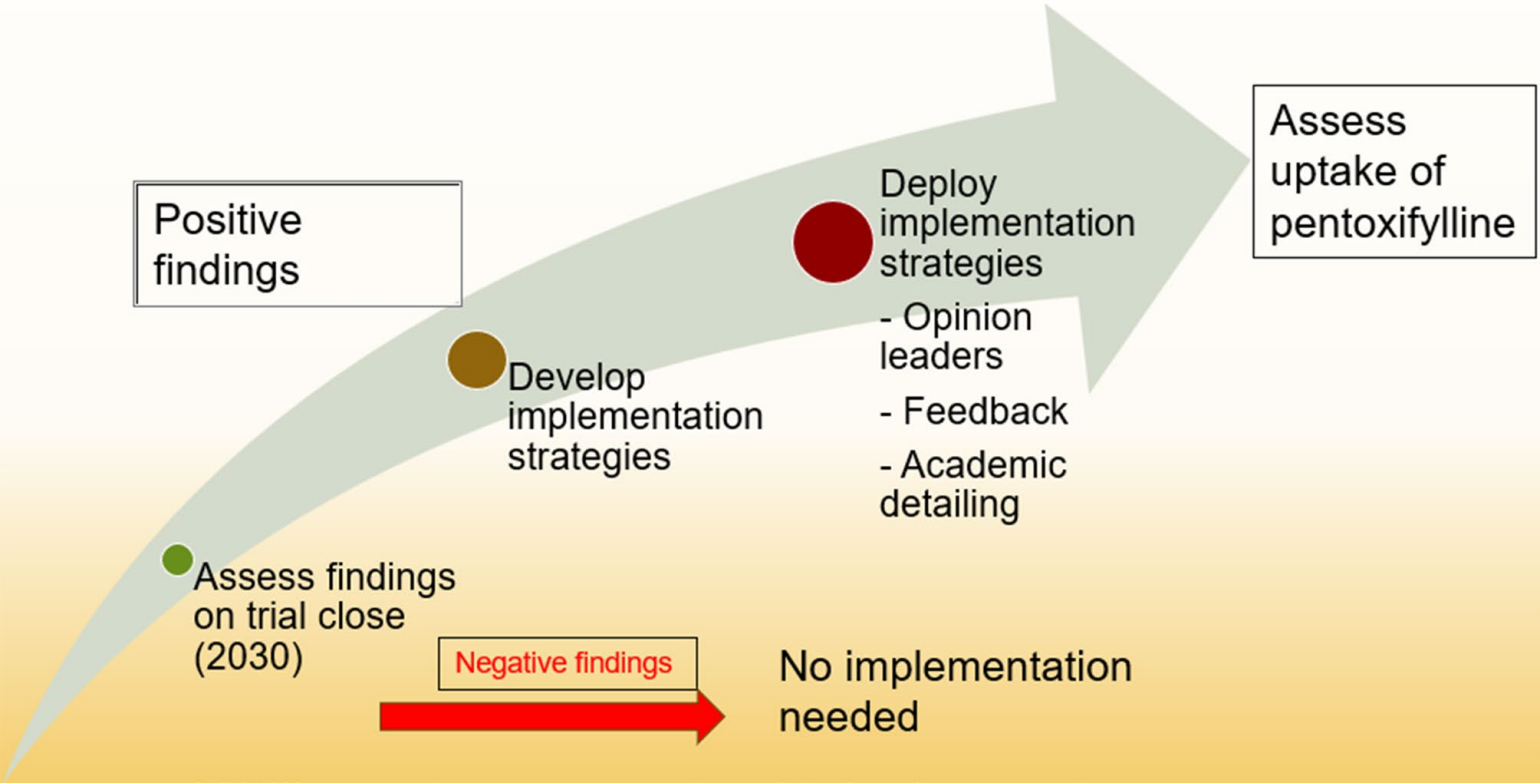
Planning for implementation

Our implementation science team aims to develop a comprehensive VA-wide implementation plan by focusing on key implementation outcomes, identifying and interviewing key stakeholders, and identifying barriers and facilitators to implementing findings if the findings support the hypotheses. In the context of an ongoing clinical trial, focusing on identifying barriers and facilitators allows for rapid identification of actionable determinants that can directly inform the design of implementation strategies. We aim to support implementation of trial findings and will tailor implementation interventions to different VA facilities and contexts, including different types of providers (Fig. 1: Planning for Implementation).

Though changing prescribing behavior is relatively simple, there is ample evidence that many factors may impede change. Some of these factors include drug familiarity, influence from colleagues, and current practice at a local clinic or hospital [13]. Organizations often need to deploy multiple implementation strategies to successfully implement an innovation [14]. By ascertaining barriers and facilitators to prescribing pentoxifylline, we will plan multiple implementation strategies for use across VA nationally and potentially in non-VA healthcare organizations. We conducted a mixed methods exploratory study, with qualitative interviews followed by a quantitative survey, developing the survey based on findings from the interviews and asking respondents to rank identified barriers by perceived importance.

## Qualitative methods

### Participants and recruitment

We provide a brief overview of methods here, with further detail in Additional File 1. CSP 2008 initially recruited participants from 6 VA pilot sites to test trial procedures, including recruitment and central drug distribution [9]. We conducted initial qualitative interviews with staff from the pilot sites, and then expanded to include an additional 4 VA facilities. We conducted thirty-three interviews between June 2022 and September 2023.

### Data collection

We developed a semi-structured interview guide with input from the clinical trial team (see Additional File 2: Interview Guide). Topics included attitudes and beliefs toward pentoxifylline use in patients with diabetic kidney disease, comfort with off-label prescribing, and factors that influence choice in prescribing a medication. Each interview was conducted using Microsoft Teams and included both an interviewer and a dedicated notetaker.

This study was deemed exempt by the VA Central IRB and the Ann Arbor Research and Development Committee under Exemption Category 2 of the Revised Common Rule, with a waiver of written informed consent. Interviews lasted between 13 and 35 minutes, with the average length being 20 minutes. We used the ASSESS tool as the reporting guideline (Additional File 3).

### Data analysis

We used an adapted rapid analytic approach to analyze the qualitative interviews [15, 16]. Interviews were analyzed using the Tailored Implementation in Chronic Disease (TICD) framework [17], which was selected because it’s the most recent, consolidated determinants framework. The TICD incorporates key aspects of 12 previous frameworks or models, including the Consolidated Framework for Implementation Research (CFIR) and the Theoretical Domains Framework (TDF). The TICD consists of 57 determinants organized in 7 domains that might impact successful implementation of innovations. Given our goal of prospectively identifying potential influences on prescribing behavior across a variety of provider types, the TICD offered a sufficiently broad sample of determinants.

The dedicated notetaker (AR) served as the primary analyst on each interview and the interviewer (JK) served as the secondary analyst and created brief summaries of the interview data. To manage the breadth of the TICD framework, analysts used a structured coding template and had iterative consensus discussions to ensure consistent application of constructs. Analysts sorted quotes into TICD domains and constructs and grouped by barriers and facilitators. We used MAXQDA for qualitative data management to support analysis.

## Quantitative methods

### Survey development

We developed the survey based on interview findings, focusing on topics that were frequently discussed by interview participants. The survey instrument is attached as Additional File 4. We asked survey respondents about their awareness of the Pentoxifylline in Diabetic Kidney Disease trial (CSP 2008), their ranking of the barriers identified through interviews, and their methods of obtaining information about prescribing drugs to VA patients.

### Data analysis

Survey data were analyzed using REDCap, which provides built-in tools for generating descriptive statistics. Given our objective of integrating qualitative and quantitative findings, descriptive statistics were sufficient for summarizing survey results and assessing concordance with qualitative insights.

## Results

### Qualitative findings

The analytic team identified five key facilitators and five potential barriers to prescribing pentoxifylline should the trial demonstrate effectiveness of pentoxifylline. These five facilitators and barriers reflect thematic aggregation of frequently observed determinants across interviews, rather than a one-to-one mapping to individual constructs. The most commonly used TICD domains included individual health professional factors, incentives and resources, and professional interactions. In Table 1, we provide exemplar quotations from the interviews, with their associated TICD domains and constructs.

**Table 1:** Exemplar quotations from interviews

TICD Domain	Construct	Respondent	Quotation
**Facilitators (Themes)**			
*Lack of concern prescribing an older medication*			
*Individual health professional factors*	*Agreement with recommendations*	Nephrologist	*“In fact, I would feel more comfortable prescribing an older drug because it has a longer track record. If something adverse were to be associated with it, I think we would know by now …”*
*Individual health professional factors*	*Intention and motivation*	Primary care provider	*“… I think that actually makes it more comfortable because it’s well known as far as its side effect profile and it’s more studied. I feel more comfortable with it because I remember using it early in my career.”*
*Lack of concern about side effects*			
*Individual health professional factors*	*Agreement with recommendations*	Pharmacist	*“I’m always a fan of our older medications because I think there’s so much more information about the side effect profile than you would see with the newer agent.”*
*Individual health professional factors*	*Expected outcome*	Pharmacist	*“But as far as the drug itself, pentoxifylline, I’ve never anecdotally never had a patient come to me and say they’re having a side effect or see a real side effect related to it. So the safety of the drug is pretty good.”*
*Pentoxifylline is considered inexpensive*			
*Incentives and resources*	*Financial incentives and disincentives*	Pharmacist	*“I feel like older drugs are usually pretty cost effective because they are not currently patented so that’s always a big plus.”*
*Incentives and resources*	*Financial incentives and disincentives*	Pharmacist	*“Yeah, if the cost is significantly cheaper than other drugs that we give then yes, it would be definitely a factor.”*
*Widespread comfort with off-label prescribing*			
*Individual health professional factors*	*Attitudes towards guidelines in general*	Pharmacist	*“I think there are lots of useful drugs out there that have been around for a long time and maybe not necessarily funded by pharma to be studied for a specific indication, but nonetheless still potentially beneficial. So, I definitely use medications off label on a regular basis.”*
*Individual health professional factors*	*Attitudes towards guidelines in general*	Nephrologist	*“[I am] relatively comfortable prescribing off-label if I have personal experience and a general practice pattern of my colleagues and there is clinical practice guideline recommendations.”*
*Providers would prescribe if trusted colleagues recommended it*			
*Professional interactions*	*Communication and influence*	Nephrologist	*“I definitely take my cues from colleagues, especially those with more experience. I have colleagues who are more experienced clinically, but I also have colleagues who are not necessarily clinically experienced but know the scientific literature better. So, both of those groups are influential to me”*
**Barriers (Themes)**			
*Concerns about drug interactions*			
*Individual health professional factors*	*Intention and motivation*	Nephrologist	*“And I think unless the evidence is very, very, very strong, and it’s rarely strong in situations like this, where background therapy is so effective. So, what you end up looking at is maybe an incremental improvement and I think it’s going to be a hard sell to introduce an additional twice-a-day medication for an incremental improvement where the prospects for drug/drug interactions can’t be known.”*
*Many providers self-identify as late adopters*			
*Individual health professional factors*	*Knowledge about own practice*	Primary care provider	*“I like to wait. I don’t jump right away. Even though there is evidence, trials only can go so far. Real life is different when it’s applied to millions and so I do want to wait and see how the post-market data is.”*
*Concerns about polypharmacy*			
*Patient factors*	*Patient behavior*	Endocrinologist	*“A huge problem at the VA and probably everywhere else is polypharmacy. So, you can sometimes see a patient who’s on 25 to 30 medicines.”*
*Patient factors*	*Patient needs*	Primary care provider	*“I think it is a good idea, but also polypharmacy is definitely, I mean I need to see the evidence that it is a good thing before adding one more medicine to the list.”*
*Preference for newer drugs*			
*Guideline factors*	*Compatibility*	Nephrologist	*“Also, all these patients are at this point all on GLP1s too. You’ll see these medications referred to nowadays as the pillars of treating diabetic nephropathy … there may end up being too many pillars.”*
*Individual health professional factors*	*Agreement with recommendations*	Pharmacist	*“And so that would be my little worry with, I guess kidney disease or diabetic kidney disease is we have an arsenal of drugs already in our ACEs, ARBs, SGLT2s, GLP1s. What would be the role for this product?”*
*Providers want ample evidence that pentoxifylline is effective*			
*Guideline Factors*	*Strength of recommendation*	Primary care provider	*“I would probably want to see randomized data and again, could you sway me if you have a very strongly positive study in the VA and the VA marketed it? Yes. Ordinarily, would my practice change after a single RCT? No. I probably would want to see more multiple RCTs, or even waiting for guidelines.”*

#### Facilitators

##### Providers are not concerned about prescribing an older medication

Despite pentoxifylline being an older medication, providers viewed this as an advantage rather than a concern. They highlighted the well-documented safety profile, which increases their confidence in prescribing.

##### Providers are not concerned about side effects of pentoxifylline

Providers shared that the side effect profile of a medication strongly influences their prescribing decisions. The side effect profile for pentoxifylline is relatively benign with gastrointestinal symptoms being the most common, though these typically resolve over time [18]. Since the medication has been on the market so long, the limited side effects are known and can be explained to patients prior to prescribing. The dosage of pentoxifylline that is being used in CSP 2008 is low, which providers believe will also decrease the amount of side effects.

##### Pentoxifylline is considered inexpensive

Cost is a key factor in medication selection. Because pentoxifylline is an older medication and generic, the drug is viewed as an inexpensive option. Though costs to the patient in the VA system are not as burdensome as in the private sector, affordability could support broader implementation beyond the VA. VA facilities bear most of the costs of drugs provided to patients through their pharmacy budgets [19, 20].

##### Widespread comfort with off-label prescribing

After a drug is approved by the US Food and Drug Administration (FDA) clinicians can prescribe the drug for treatments not specified on the approved label, which is considered off-label use [21]. Providers expressed confidence in prescribing medications off-label, particularly when supported by clinical experience and peer recommendations. Providers stated that they need evidence that an off-label drug is beneficial, but do not require an FDA approval.

##### Providers would prescribe if trusted colleagues recommended it

Providers shared that they become comfortable prescribing a medication if their trusted colleagues recommend it. These providers may be specialists for a given condition or colleagues with a robust understanding of the scientific literature. These conversations are sometimes ad-hoc but also occur during team meetings, journal clubs and grand round presentations.

Across facilitators, a consistent pattern emerged in which clinical acceptability of pentoxifylline seemed to be reinforced by social and experiential factors. Providers expressed comfort prescribing an older medication for an off-label indication, particularly when endorsed by respected peers. This finding suggests that social influences could be leveraged to amplify favorable perceptions of pentoxifylline.

### Barriers

Several potential barriers to prescribing pentoxifylline emerged, which will need to be addressed in future dissemination and implementation efforts, including implementation strategy design:

#### Concerns about drug interactions

Providers expressed concern regarding pentoxifylline’s potential interactions with other medications that are commonly prescribed for their patient population. Providers want evidence that there are few drug-drug interactions so that they will feel comfortable prescribing and be able to reassure their patients that it’s safe.

#### Many providers self-identify as late adopters

Providers were asked at what stage they become comfortable in prescribing a drug when new information is available. Though some providers self-identified as being early adopters, many providers stated that they consider themselves to be late adopters and prefer to wait for additional evidence before prescribing.

#### Concerns about polypharmacy

Providers explained that most of their patients are already taking many medications for their comorbidities and are concerned about polypharmacy. Providers want to know the role and benefit of pentoxifylline given the fact that it will be an additional medication. They also voiced concern about needing to coordinate with other providers to make sure pentoxifylline won’t interact with drugs prescribed by other providers.

#### Preference for newer drugs

When asked their thoughts about prescribing pentoxifylline for patients with diabetic kidney disease, providers often spoke about the widespread use of sodium-glucose cotransporter 2 (SGLT2) inhibitors and glucagon-like peptide-1 (GLP-1) receptor agonist drugs. They stated that the evidence has been so strong, and the renal benefit so profound that the VA has initiatives to promote prescribing SGLT2 inhibitors for this population despite the high cost [22]. Providers want to understand whether pentoxifylline adds value above and beyond the medications that are currently being used for treatment.

##### Providers want ample evidence that pentoxifylline is effective

The providers we spoke with stated that they will want ample evidence that pentoxifylline is effective for treating patients with diabetic kidney disease prior to prescribing. Some providers stated that they would like data from more than one randomized controlled trial, and others stated that they would prefer to wait until more is known when the drug is being prescribed in real world conditions outside of a trial, or once guidelines are released endorsing its use.

Interviewee awareness of the clinical trial prior to participating in the qualitative interview was variable. Interview responses highlighted that 43% of interviewees who were not directly involved in the clinical trial at their VA facility were unaware that the trial was taking place. Pharmacists and primary care physicians were less aware than nephrologists.

## Quantitative findings

### Awareness of CSP 2008 trial

Providers were asked whether they were aware of CSP 2008 prior to being sent the survey invitation (Table 2). Altogether, 66% of respondents indicated they were not familiar with CSP 2008 prior to being asked to complete the survey. This finding varied by provider type: 89% of PCPs were unfamiliar, 84% of pharmacists, 73% of endocrinologists and 15% of nephrologists were unfamiliar.

**Table 2:** Respondent specialty and awareness of CSP 2008

	N	%
**Respondent specialty**		
Nephrologist	33	23.9
Endocrinologist	15	10.9
Primary Care Provider	37	26.8
Pharmacist	45	32.6
Other	8	5.8
**Aware of CSP 2008 prior to survey**		
Yes	47	33.8
No	92	66.2

### Facilitators

Survey responses aligned with qualitative findings in some areas but varied in others (Table 3). Only 18.8% of respondents considered prescribing an older medication to be a moderate or extreme concern. Two perceived facilitators during the qualitative interviews that had variable survey responses included: being uncomfortable with off-label prescribing (43.1%) and lack of concern about the safety profile of pentoxifylline (40.4%).

**Table 3:** Survey responses

	Not at all important, n (%)	Slightly important, n (%)	Neutral, n (%)	Moderately important, n (%)	Extremely important, n (%)
Concerned about polypharmacy	5 (3.6)	18 (13.0)	25 (18.1)	58 (42.0)	32 (23.2)
Concerned about limited RCT evidence	3 (2.2)	10 (7.4)	35 (25.7)	48 (35.3)	40 (29.4)
SGLT2 inhibitors are my top pick for treating diabetic kidney disease	0 (0.0)	4 (3.0)	30 (22.2)	57 (42.2)	44 (32.6)
Identify as a “late adopter” for prescribing	27 (20.6)	13 (9.9)	71 (54.2)	16 (12.2)	4 (3.1)
Have questions regarding how pentoxifylline interacts with other drugs	6 (4.3)	18 (13.0)	35 (25.4)	53 (38.4)	26 (18.8)
Uncomfortable prescribing drugs off-label	14 (10.2)	28 (20.4)	36 (26.3)	46 (33.6)	13 (9.5)
Concerned about the safety profile of pentoxifylline	10 (7.4)	20 (14.7)	51 (37.5)	46 (33.8)	9 (6.6)
Concerned about prescribing an older medication	46 (33.3)	25 (18.1)	41 (29.7)	20 (14.5)	6 (4.3)
Concerned about 2x a day prescribing	26 (18.8)	41 (29.7)	33 (23.9)	34 (24.6)	4 (2.9)

### Barriers

The survey results confirmed that polypharmacy is a major concern for providers with 65.2% of respondents stating concerns about polypharmacy are moderately to extremely important. Being concerned about limited randomized controlled trial evidence was indicated by 64.7% of respondents. Aligned with the qualitative findings, SLGT2 inhibitors were identified as the top choice for treating diabetic kidney disease for 74.8% of survey respondents. Questioning how pentoxifylline interacts with other medications was a concern for 57.2% of respondents. Only 15.3% of survey respondents identified as being late adopters for prescribing new medications when guidance changes, which differed from the qualitative findings.

### New prescribing recommendations

To assist with creating a dissemination and implementation plan, we wanted to know how providers are currently learning about new prescribing recommendations and their preferred way for learning about new prescribing recommendations (Table 4). Providers report most commonly receiving prescribing updates from their local VA facility (53.6%) and their specialty service group (57.2%). When asked about their preferred single source for learning about new recommendations, 34.8% preferred their specialty service group, with the remainder split across the other sources.

**Table 4:** Prescribing recommendations

How do you usually learn about new prescribing recommendations for medications?	N	%
National VA	55	39.9
PBM	42	30.4
Local VA Facility	74	53.6
P&T Committee	35	25.4
Your specialty service group	79	57.2
Other	34	24.6
**How you prefer to learn about new prescribing recommendations for medications**	N	%
National VA	19	13.8
PBM	15	10.9
Local VA Facility	27	19.6
P&T Committee	11	8
Your specialty service group	48	34.8
Other	18	13

## Discussion

In this mixed methods study embedded within an ongoing clinical trial, we identified key barriers and facilitators that may influence the uptake of pentoxifylline for diabetic kidney disease if trial results demonstrate effectiveness. Our use of the TICD systematically informed our work by providing a structured lens to identify determinants of provider behavior relevant to prescribing pentoxifylline. Use of the TICD helped clarify how facilitators and barriers operate at multiple levels, offering targets for tailoring implementation strategies [23].

Across findings, patterns emerged that provide insight into how providers may respond if pentoxifylline is found to be effective for treating diabetic kidney disease. The facilitators appeared to reinforce one another, with providers expressing comfort with prescribing older medications for off-label indications, particularly when reinforced by the endorsement of respected peers [24]. Peer influence emerged as a central facilitator which can be leveraged by implementation strategies such as an opinion leader intervention, which could be deployed at the national or facility level in VA [25–27]. We can begin to plan for this implementation strategy by identifying leaders at facilities who are regarded as influential by their colleagues [28, 29].

We identified several potential barriers that reflected a more cautious and evidence-based approach to prescribing. Concerns about polypharmacy, drug interactions and the need for robust evidence were all consistently raised. Pentoxifylline has been prescribed since the 1980s, and our results indicate that providers are not concerned with using an older medication. As an older medication, it is relatively inexpensive, and much is known about the long-term safety profile. Providers did express a preference for using newer medications which appeared to be in tension with the acceptability of using older medications. We anticipate being able to leverage the benefits of using a lower cost medication with a well-known safety profile to influence prescribing.

Taken together, our findings suggest a coherent portrait of the VA provider as an implementer. VA providers appeared to be relatively conservative, cost-conscious and strongly influenced by peer consensus. These findings have important implications when choosing and designing implementation strategies. Providing clear accessible evidence and leveraging peer endorsement, such as using audit with feedback may be particularly effective. Audit with feedback is used to provide recipients with summaries of their performance or behaviors [30]. Our team has considerable experience with audit with feedback and can begin the user-centered design process as the trial progresses [31, 32]. Feedback could be a standalone strategy or coupled with academic detailing, which is commonly used in the VA to promote changes in prescribing practices and uses specially trained clinical pharmacists who promote prescribing best practices [33].

While the specific barriers and facilitators identified in this study are shaped by the VA context and the ongoing clinical trial, the approach used here is transferable to other implementation efforts. Embedding rapid qualitative interviews and survey methods into an ongoing clinical trial, guided by a comprehensive determinants framework, allows for the early identification of actionable factors that can inform implementation strategy development. This approach is particularly valuable when timely uptake of evidence is a priority.

## Conclusions

By prospectively identifying implementation determinants during an ongoing trial, this study demonstrates a practical approach to reduce the time between evidence generation and clinical adoption. Through our qualitative and quantitative data collection, we identified key facilitators and barriers for prescribing pentoxifylline if the trial results are positive, showing effectiveness of pentoxifylline in retarding progression of diabetic kidney disease. To optimize implementation, we will proactively leverage facilitators while addressing potential barriers. Continuing to ascertain barriers and facilitators for prescribing pentoxifylline while developing implementation strategies during the trial will facilitate the spread, adoption, and routinization of use.

Our findings suggest that successful implementation of pentoxifylline will depend not only on demonstrating effectiveness, but on leveraging social influence, addressing concerns about polypharmacy and drug interactions, and providing ample evidence to providers.

This study contributes to the growing body of literature about the importance of planning for dissemination and implementation as a clinical trial is being conducted. It may provide insights into important questions of how to accelerate the uptake of new research evidence into practice [34].

## Electronic supplementary material

Below is the link to the electronic supplementary material.

Supplementary Material 1

Supplementary Material 2

Supplementary Material 3

Supplementary Material 4

## Data Availability

Data will be available from the authors on request.

## Abbreviations

CFIR: Consolidated Framework for Implementation Research
CSP: Cooperative Studies Program
CSP 2008: Pentoxifylline in Diabetic Kidney Disease study
ORD: Office of Research and Development
PBM: Pharmacy Benefits Management
TDF: Theoretical Domains Framework
TICD: Tailored Implementation in Chronic Disease framework
VA: Department of Veterans Affairs
