# Supplementary material for: Anticipating implementation: A mixed methods study of future prescribing determinants within an ongoing trial of clinical effectiveness
Source: Implement Sci Commun. 2026 May 29;7:145. doi: 10.1186/s43058-026-00969-0 (PMC13455494; doi:10.1186/s43058-026-00969-0)
Supplement: Supplementary file 1 [file 43058_2026_969_MOESM1_ESM.docx]

**Additional File 1: Detailed Methods**

**Qualitative**

*Recruitment*:

Nephrologists, primary care physicians, pharmacists, members of the Pharmacy and Therapeutics Committees and members of the local CSP 2008 study team at each site were sent recruitment emails asking them to participate in a brief interview. The recruitment email included a participant information sheet and a copy of the interview questions. Potential interviewees were identified using publicly available email listservs and individuals were randomly selected and invited to participate.

*Approvals:*

We received VA national union concurrence to conduct the staff interviews. Prior to each interview, we obtained verbal consent from participants to participate in the interview and to audio record.

**Quantitative**

*Participants and Recruitment*

We invited nephrologists, endocrinologists, pharmacists and primary care physicians from 15 VA Medical Centers enrolling Veterans in CSP 2008 to complete a survey in REDCap. Providers were randomly selected using email listservs for the specific provider groups targeted. Providers were sent up to three emails and were also messaged via Microsoft Teams to remind them to complete the survey. A total of 450 providers were invited, with 139 surveys completed, yielding a 30% response rate.

*Data collection*

Providers were asked to rank the identified barriers and facilitators from the interviews on a 5-point Likert scale from not at all important to extremely important (see Additional File 4: Survey). The survey was deemed exempt by the VA Central IRB and the Ann Arbor Research and Development Committee under Exemption Category 2 of the Revised Common Rule and VA national union concurrence was obtained prior to survey administration.
