# Supplementary material for: Anticipating implementation: A mixed methods study of future prescribing determinants within an ongoing trial of clinical effectiveness
Source: Implement Sci Commun. 2026 May 29;7:145. doi: 10.1186/s43058-026-00969-0 (PMC13455494; doi:10.1186/s43058-026-00969-0)
Supplement: Supplementary file 2 [file 43058_2026_969_MOESM2_ESM.docx]

**Additional File 1 File: Interview Guide**

**Questions**

1. First, what is your position at (VA site)?
2. What is your experience treating Veterans with diabetic kidney disease?
3. Are you aware of and familiar with CSP 2008? (randomized clinical trial that is testing the hypothesis that pentoxifylline, when added to usual care, leads to a reduction in the incidence of end-stage renal disease and mortality in type 2 diabetic patients with diabetic kidney disease)
4. What is your understanding of why pentoxifylline is being studied?
5. What are your thoughts about prescribing Pentoxifylline for your patients with diabetic kidney disease?
   a. Probe: Do you have any concerns about safety (retinal hemorrhage, GI side effects), using of an older drug, competing drugs (GLP1, SGLT2 inhibitors), costs, patient burden or drug adherence (another medication, twice a day), other?
6. What individuals, organizations, guidance bodies, or norms/values do you rely on for guidance on how you treat Veterans with diabetic kidney disease?
   a. Probe: How do you stay current on scientific advancements in treatments for diabetic kidney disease?
7. At what stage do you become comfortable in prescribing a drug when new information is available?
   a. Probe: after a rigorous clinical trial shows safety and effectiveness; after FDA approves a drug for a specific indication; after a clinical body issues a formal recommendation for use; once a drug becomes generic due to cost, etc.)
8. How comfortable are you prescribing drugs “off-label”? What type of data or guidance would convince you that off-label prescribing of a specific drug may be advantageous for your patients?
9. What are some factors that influence your choice in prescribing a drug for a given patient, particularly as new drugs are approved or more data become available on older drugs?
10. If pentoxifylline is found to be effective in reducing the incidence of end-stage renal disease and mortality for type 2 diabetic patients with diabetic kidney disease, how do you think the VA should disseminate this information to providers?
11. Who are 3 influential people you turn to for guidance about treating Veterans with diabetic kidney disease?
12. Are there any issues we should talk about that haven’t come up yet?
