# Supplementary material for: Anticipating implementation: A mixed methods study of future prescribing determinants within an ongoing trial of clinical effectiveness
Source: Implement Sci Commun. 2026 May 29;7:145. doi: 10.1186/s43058-026-00969-0 (PMC13455494; doi:10.1186/s43058-026-00969-0)
Supplement: Supplementary file 3 [file 43058_2026_969_MOESM4_ESM.docx]

CSP 2008 Sub-Study Provider Survey

1. Prior to receiving this survey were you familiar with the Cooperative Studies Program, CSP 2008 “Pentoxifylline in Diabetic Kidney Disease” study that is enrolling Veterans at your VA?
   a. Yes
   b. No
2. Please select your provider type:
   a. Nephologist
   b. PCP
   c. Pharmacist
   d. Other: write in
3. We have conducted interviews with Nephrologists, PCPs and Pharmacists at sites that are participating in CSP 2008 and have learned several barriers to prescribing pentoxifylline for Veterans with diabetic kidney disease. Please rank these potential barriers in importance (from least important to most important).
   a. Concerned about polypharmacy
   b. Concerned about limited RCT evidence
   c. SGLT2 inhibitors are my top pick for treating diabetic kidney disease
   d. Identify as a “late adopter” for prescribing
   e. Have questions regarding how pentoxifylline interacts with other drugs
   f. Uncomfortable prescribing drugs off-label
   g. Concerned about the safety profile of pentoxifylline
   h. Concerned about prescribing an older medication
   i. Concerned about 2x a day prescribing
   j. Other: write in
4. How do you usually learn about new prescribing recommendations for medications? (select all that apply)
   a. National VA
   b. PBM
   c. Local VA facility
   d. P&T Committee
   e. Your specialty service group
   f. Other: write in
5. Pick which way you prefer to learn about new prescribing recommendations for medications.
   a. National VA
   b. PBM
   c. Local VA facility
   d. P&T Committee
   e. Your specialty service group
   f. Other: write in
6. If you have questions regarding treating Veterans with diabetic kidney disease, please name who you would go to for advice.
   a. Open-ended response
